# Relationships Between Nurses' Personal and Professional Characteristics and Career Decision Regret, Occupational Stress and Turnover Intention: A Descriptive Cross‐Sectional Study

**DOI:** 10.1111/ijn.70040

**Published:** 2025-08-04

**Authors:** Şehrinaz Polat, Aslı Yeşil

**Affiliations:** ^1^ Istanbul University, Faculty of Nursing Istanbul Türkiye; ^2^ Faculty of Humanities and Social Sciences, Department of Psychology Bursa Technical University Bursa Türkiye

**Keywords:** decision regret, hospital, nursing, occupational stress, turnover

## Abstract

**Aims:**

This study aimed to (a) examine the relationship between perceived career decision regret and turnover intention among nurses and (b) explore the mediating role of occupational stress and career decision regret in this relationship within the context of hospital‐based nursing practice.

**Design:**

A descriptive cross‐sectional study.

**Methods:**

Data were collected between 1 November 2023 and 20 February 2024, from a final sample of 512 nurses employed across various hospital settings in Türkiye. Standardized questionnaires measured career decision regret, occupational stress and turnover intention. Ethical approval was obtained from the institutional ethics committee, and informed consent was obtained from all participants. Group comparisons were performed using independent samples *t* tests and one‐way analysis of variance (ANOVA). Pearson correlation analysis assessed relationships between continuous variables. The mediating effect of occupational stress was evaluated using the bootstrap method at a 95% confidence interval, with statistical significance set at *p* < 0.05.

**Results:**

Nurses working in public hospitals reported significantly higher levels of occupational stress, career decision regret and turnover intention compared to those in private hospitals. Career decision regret had a direct positive effect on turnover intention (*β* = 0.37) and an indirect effect mediated by occupational stress (*β* = 0.33).

**Conclusion:**

Career decision regret directly and positively influences turnover intention, with occupational stress serving as a significant mediator. These results have important implications for the development of targeted interventions aimed at reducing occupational stress, particularly among nurses experiencing career decision regret, to improve retention and reduce turnover intentions.

**Reporting Method:**

This study adhered to the STROBE guidelines.

**Patient or Public Contribution:**

There was no patient or public involvement in this study.

## Summary

**What is already known about this topic?:**

- High turnover rates and increasing turnover intention among nurses continue to challenge healthcare systems worldwide.
- However, not all variables influencing turnover intention—whether directly or indirectly—have been fully identified, and the specific relationships that most effectively reduce turnover intention remain unclear.

**What this paper adds?:**

- This study demonstrates that occupational stress mediates the relationship between career decision regret and turnover intention among nurses.
- In addition, career decision regret mediates occupational stress that is stronger than the mediating effect of career decision regret.
- Strategies aimed at reducing occupational stress, particularly for nurses experiencing career decision regret, are crucial for reducing turnover intention.

**Implications of this study:**

- Managers and researchers should consider the relationship between career decision regret and occupational stress when designing targeted interventions to prevent or reduce nurse turnover.
- Addressing both career decision regret and occupational stress is essential in efforts to mitigate nurses' turnover intentions.

## 1 Introduction

Individuals typically expect positive outcomes and a sense of satisfaction from their life choices. However, expectations may not always align with workplace realities, leading to negative affective responses such as career decision regret. This regret can lead to cognitive dissonance and increase the likelihood of re‐evaluating career trajectories or considering strategies to mitigate regret, including turnover intentions. While decisions with minimal impact on life may be easier to address, those that significantly affect key aspects of one's life—such as career choices—are more difficult to amend. The decision to choose a profession, in particular, has profound implications for individuals' personal and professional life. As highlighted by Budjanovcanin and Woodrow (2022), individuals may experience career decision regret due to various factors, including challenges faced during professional entry, substantial investments in vocational education and perceived financial benefits of the chosen career path. Nevertheless, despite these regrets, altering one's career trajectory or reversing previous decisions often proves difficult. A study conducted by Dyrbye et al. (2020) revealed that approximately one in seven American nurses regrets their career choice, suggesting career decision regret as a significant issue within the nursing profession.

Career decision regret may arise when the realities of the chosen profession fail to align with an individual's initial expectations or when, over time, the profession becomes incongruent with evolving personal values and long‐term goals. This misalignment often contributes to elevated levels of occupational stress. Nurses experiencing career decision regret frequently report increased psychological strain due to decreased job satisfaction and a lack of professional fulfilment. The cumulative effect of persistent occupational stress combined with unresolved regret can exacerbate turnover intentions and increase the likelihood of actual turnover behaviour. In high‐responsibility and high‐stress professions such as nursing, career decision regret and occupational stress are significant factors contributing to staff turnover. These experiences can adversely impact both the personal well‐being and professional functioning of nurses. Consequently, elevated turnover rates pose substantial challenges for healthcare institutions, leading to workforce instability, increased recruitment costs and potential declines in the quality of patient care.

This study investigates the predictive effect of career decision regret on nurses' turnover intention, with occupational stress examined as a potential mediating variable. Concurrently, it explores the predictive role of occupational stress on turnover intention, while assessing the mediating influence of career decision regret within this relationship. Existing literature suggests that career decision regret is associated with various occupational and psychological outcomes, including turnover intention, burnout, job and life satisfaction, career development and stress coping mechanisms (Akturan et al. 2025; Doğanülkü and Şeker 2023; Doğrusöz et al. 2022; Sun et al. 2023; Yam and Korkmaz 2024). However, its specific impact on turnover intention within the nursing profession remains underexplored and requires further empirical investigation. Therefore, this study aims to determine whether career decision regret serves as a significant predictor of turnover intention among nurses and to evaluate the mediating role of occupational stress in this relationship.

By clarifying the relationship between career decision regret and turnover intention, this research contributes to the existing body of knowledge and supports the development of targeted interventions at both individual and organizational levels within healthcare settings.

### 1.1 Turnover Intention

The global shortage of nurses remains a persistent and escalating concern, with projections indicating that this issue will continue in the coming years (Boniol et al. 2022). A systematic review and meta‐analysis by Wu et al. (2024) reported a global cumulative nurse turnover rate of 18%. Similarly, a recent meta‐analysis reported a global nurse turnover rate of 16% (Ren et al. 2024). Mafula et al. (2025), through their meta‐analysis, reported a combined turnover rate of 15.2% and a pooled prevalence of turnover intention of 38.4%. Turnover intention refers to an employee's expressed intention to leave their organization within a specified time frame (Lazzari et al. 2022). Elevated turnover intention is often indicative of an employee nearing the point of resignation (Bae et al. 2021). Several studies have identified turnover intention as a strong predictor of actual turnover behaviour (Ki and Choi‐Kwon 2022; Lee and Kim 2020). Importantly, turnover is not a sudden event but a gradual process that unfolds over time (Ki and Choi‐Kwon 2022), eventually resulting in resignation if unaddressed.

Nurses' turnover behaviour, alongside the broader nursing shortage, has numerous negative implications for patients, nurses and healthcare organizations (Bae 2025; Cho et al. 2023; Griffiths et al. 2023; Lasater et al. 2021; Moscelli et al. 2024; Subramony et al. 2024). A recent study by Catania et al. (2024) identified turnover intention as a critical nursing outcome indicator associated with patient mortality, estimating that a 10% increase in turnover intention corresponds to a 14% rise in inpatient mortality risk. Given these severe consequences, Labrague et al. (2020) emphasize the urgent need to identify and address the antecedents and effects of nurses' turnover intention. Although not all individuals who express turnover intention ultimately resign, high levels of turnover intention within an organization often indicate systemic issues requiring intervention.

Factors contributing to nurse turnover can be grouped into four categories: personal factors (e.g., sociodemographic characteristics such as age, education and gender and psychological aspects such as person–organization fit, career commitment, stress and burnout), professional and job‐related factors (e.g., workload, role conflict, autonomy, shift schedules, job satisfaction, stress and opportunities for career development or promotion), interpersonal factors (e.g., relationships with leaders and colleagues, workplace mobbing and lack of respect) and organizational factors (e.g., work environment and salary) (Cardiff et al. 2023; H. Kim and Kim 2021; Nam et al. 2025; O'Callaghan and Sadath 2025; Rhéaume et al. 2025). While some of these factors are rooted in pre‐existing personal characteristics (e.g., age and gender), others evolve throughout professional practice. One such factor—career decision regret—may develop both prior to and after entering the profession and has the potential to contribute significantly to turnover intention.

### 1.2 Career Decision Regret

Regret, as defined by Zeelenberg and Pieters (2007) is ‘the emotion that we experience when realizing or imagining that our current situation would have been better, if only we had decided differently. It is a backward‐looking emotion signaling an inappropriate evaluation of a decision. It is an unpleasant feeling, coupled with a clear sense of self‐blame concerning its causes and strong wishes to undo the current situation’ (p. 3).

According to Zeelenberg and Pieters' (2007) theory of regret, this emotion arises when individuals perceive that an alternative could have resulted in a more favourable outcome. Feiler and Müller‐Trede (2022) found that when individuals lack sufficient information about the outcomes of unchosen options, they tend to idealize these alternatives compared to their actual decisions, thereby intensifying feelings of regret. Matarazzo et al. (2021) further argue that regret is particularly pronounced when individuals experience limited autonomy in decision‐making, such as being compelled to make choices that contradict their personal preferences. From a professional standpoint, career decision regret is often associated with dissatisfaction, disillusionment and a poor sense of career fit (Brehaut et al. 2003; Erdurcan and Kırdok 2017). Specifically, Yam and Korkmaz (2024) define career decision regret as a negative emotional response that emerges when an individual's career choice fails to meet their initial expectations and aspirations.

Nurses' decisions to enter the profession are often influenced by a combination of intrinsic and extrinsic factors. While some individuals are intrinsically motivated by a genuine interest in healthcare or a desire to help others, external influences such as job security, socioeconomic factors, family expectations, social norms or random placement based on university entrance exam scores can also significantly shape this career choice (Çalık Bağrıyanık et al. 2023; İncirkuş et al. 2023; Öncü et al. 2022). According to Budjanovcanin and Woodrow (2022), individuals who experience career decision regret frequently report a fundamental misalignment between themselves and their chosen profession. This misalignment may manifest itself as a lack of enjoyment in the nature of the work, incompatibility with their desired lifestyle or a diminished sense of meaning and purpose. The authors further note that such regret is often linked to insufficient self‐awareness or limited professional knowledge at the time the decision was made.

While some nurses may respond to career decision regret by seeking to transition to a different profession early in their careers, others choose to remain in nursing despite experiencing regret (Budjanovcanin and Woodrow 2022). According to Zeelenberg and Pieters' (2007) theory of regret, individuals adopt various strategies over time to manage or minimize the impact of regret. However, for those unable to reverse their career decisions due to obligations such as family responsibilities, prior professional investments or economic constraints, prolonged engagement in an undesired profession may lead to negative outcomes. Although occupational regret is identified as a key mechanism influencing life events, workplace behaviours and professional outcomes (Budjanovcanin and Woodrow 2022), research specifically examining career decision regret remains limited. Previous studies have demonstrated that career decision regret is negatively associated with career adaptability, job satisfaction and life satisfaction, while positively correlated with adverse outcomes such as burnout (Akturan et al. 2025; Doganülkü and Kirdök 2021; Doğanülkü and Şeker 2023; Dyrbye et al. 2020; Sun et al. 2023; Tian et al. 2019). These variables are well‐established predictors of both turnover intention and actual employee resignation. Budjanovcanin et al. (2019) reported that among cardiac physiologists, individuals experiencing career decision regret tend to exhibit lower emotional commitment to their profession and an increased intention to leave the field. Doğanülkü and Güneşlice (2022) found that career decision regret negatively predicts proactive career behaviours, suggesting that the dissatisfaction, frustration and burnout associated with regret may reduce optimism and impede engagement in future‐oriented actions. Similarly, Yam and Korkmaz (2024) reported that career decision regret adversely affects psychological well‐being, professional outcome expectations and proactive behaviours. Furthermore, career decision regret has been consistently found to negatively correlate with career adaptability (Bilgiz‐Öztürk and Karabacak‐Çelik 2023; Doganülkü and Kirdök 2021; Doğanülkü and Şeker 2023) and serves as a significant predictor of burnout (Doganülkü and Kirdök 2021; Dyrbye et al. 2020) and lower job satisfaction (Akturan et al. 2025). These variables are well‐established contributors to turnover intention and actual resignation (Akturan et al. 2025; O'Callaghan and Sadath 2025; Sun et al. 2023; Zhang et al. 2025; Zheng et al. 2025). Budjanovcanin et al. (2019) also noted that individuals who experience career decision regret tend to exhibit lower emotional commitment to their profession alongside higher turnover intention. To date, only one study has specifically examined the relationship between career decision regret and turnover intention among nurses, confirming that career decision regret significantly and positively predicts nurses' turnover intention (Doğrusöz et al. 2022). Given the adverse outcomes associated with career decision regret and turnover, it is crucial to develop and implement strategies aimed at mitigating or managing career decision regret.

Festinger's (1957) cognitive dissonance theory (CDT) suggests that cognitive dissonance generates psychological discomfort, which motivates individuals to engage in dissonance reduction strategies aimed at restoring cognitive consistency. To resolve dissonance, individuals may modify their behaviour, alter existing cognitions, adopt new compatible beliefs or disregard information that intensifies the dissonance (Festinger 1957; Hinojosa et al. 2017). Applying CDT to nursing, cognitive dissonance may arise when nurses find their profession misaligned with personal expectations and values, contributing to increased occupational stress. To alleviate this dissonance, nurses may seek behavioural changes, such as transitioning to a different profession or developing cognitions that justify turnover intentions until psychological relief is achieved. From the perspective of the theory of regret (Zeelenberg and Pieters 2007), regret prompts individuals to re‐evaluate their decisions and consider alternative career paths. For nurses, this may involve exploring roles or workplaces that better align with personal and professional goals; this may serve as a coping mechanism to alleviate the negative emotions associated with career decision regret. The hypotheses are as follows:

*Career decision regret affects turnover intention among nurses*.

*Occupational stress affects turnover intention among nurses*.

Dyrbye et al. (2020) reported that frequent unscheduled or mandatory overtime work in the past month significantly predicts career decision regret. Similarly, a study conducted in the service sector found that exposure to workplace rudeness is associated with increased levels of career regret (Shin et al. 2025). According to Budjanovcanin and Woodrow (2022), occupational regret often follows a cyclical or episodic pattern, with a variety of causes and triggers. These triggers are linked to work‐related factors (e.g., characteristics of the work environment, high job stress, long and unpredictable working hours, job loss, workplace bullying, unsatisfactory job experiences, missed promotions and unfavourable workplace comparisons). However, regret may also be triggered by non‐work‐related events, including personal crises (Budjanovcanin and Woodrow 2022). A study conducted among healthcare professionals during the COVID‐19 pandemic identified sources of stress as key factors triggering regret (Park et al. 2023). Furthermore, acute stress has been shown to intensify both the experience of regret and anticipatory emotions (Wu et al. 2021). Research involving physicians demonstrated that elevated levels of stress, depression and anxiety are significantly associated with career decision regret, highlighting that stressors such as heavy workload and emotional challenges exacerbate individuals' career decision regret (Emiral et al. 2024). Akturan et al. (2025) reported that occupational stress has a significant positive effect on career decision regret among nurses. These findings indicate that as stress levels increase, the likelihood and intensity of career decision regret rise significantly. Nurses may experience career decision regret either early in their careers—at the point of entry into the profession—or later, in response to cumulative challenges encountered during professional practice. Given the inherently stressful and demanding nature of the nursing work environment, occupational stress may play a critical role in the development of career decision regret.

*Career decision regret has a significant effect on occupational stress among nurses*.

On the other hand, career decision regret is conceptualized as a negative emotional response (Brehaut et al. 2003; Erdurcan and Kırdok 2017). Prior research suggests that negative emotional states in professional settings are positively and significantly correlated with occupational stress, turnover intention and burnout, while positive emotional experiences tend to mitigate these adverse outcomes (Cetin Aydın et al. 2021). According to Budjanovcanin and Woodrow (2022), the initial catalyst for experiencing professional regret—referred to as the ‘origin trigger’—may occur as early as during an individual's professional training. Following this origin trigger, episodic triggers arising throughout an individual's career may repeatedly evoke experiences of professional regret. From this perspective, a nurse may experience occupational regret upon entering the profession, encounter it for the first time after starting work and subsequently face recurrent episodes of regret triggered by various work‐related factors. Career decision regret thus represents a persistent negative impact, with ongoing cognitive dissonance remaining salient when such regret frequently arises in a nurse's reflections. Persistent cognitive engagement with perceived negative outcomes—such as repetitive rumination and retrospective questioning—may intensify maladaptive thought patterns and emotional distress. This decision regret can deplete cognitive resources and impair psychological resilience, thereby contributing to increased occupational stress. While occupational stress may act as an antecedent to the development of career decision regret, regret itself may also serve as a stress‐inducing factor, compounding the psychological burden experienced by nurses. In other words, elevated occupational stressors may precipitate career decision regret, which in turn can amplify nurses' perception of existing stressors, creating a self‐reinforcing cycle. H2b was formulated on the premise that occupational stress and career decision regret among nurses interact dynamically, exerting reciprocal influences on each other.

*Occupational stress has a significant effect on career decision regret among nurses*.

### 1.3 Occupational Stress

Leka et al. (2003) defines occupational stress as ‘the response people may have when presented with work demands and pressures that are not matched to their knowledge and abilities and which challenge their ability to cope’. Within a similar framework, Gunasekra and Perera (2023) conceptualize occupational stress as a psychological reaction to pressures or demands inherent in one's professional responsibilities (Gunasekra and Perera 2023). It is widely recognized as a global occupational health issue, closely associated with both acute and chronic health problems, impaired job performance and a broad range of psycho‐emotional difficulties (Kasidouli et al. 2024).

In the literature, occupational stress has been examined as a predictor of various individual and organizational outcomes and as a mediating or moderating variable within complex relationships among other factors. The causal relationships between stressors, stress responses and resulting symptoms are multifaceted and multidimensional (Almino et al. 2024). Nursing is identified as one of the most demanding and high‐risk professions worldwide (Norful et al. 2024). A recent study by Zhong et al. (2025) highlighted various occupational stressors affecting nurses, including excessive workload, time constraints, inadequate compensation, perceived low professional status, irregular shift patterns, administrative burdens and the persistent fear of clinical errors. Similarly, Şanlıtürk (2021) reported that extended working hours, disproportionate nurse‐to‐patient ratios, heavy workloads and concerns regarding treatment outcomes are key contributors to occupational stress among nurses. Lee and Kim (2020) identified stressors related to patients and their families, workload, conflicts with supervisors and conflicts with peers as significantly associated with hospital nurses' turnover intention. Saifan et al. (2024) emphasize the profound emotional stress experienced by oncology nurses, which is intensified by recurring exposure to patient mortality, disease progression and illness recurrence, factors that collectively undermine nurses' psychological well‐being in oncology settings. Additional risk factors contributing to occupational stress include time constraints, limited autonomy over work‐related decisions, extended working hours, rotating shifts, insufficient institutional support and moral injury (Meneguin et al. 2024). Similarly, Kaihlanen et al. (2023) reported that increased time pressure, role conflict and wage reductions are associated with elevated stress levels among nurses. Deceptive or misleading work expectations and excessive workloads are significant contributors to occupational stress, alongside poor collegial relationships and low professional recognition (Shrivastava et al. 2024). In addition, organizational factors such as participation in decision‐making processes, team cohesion and shift work patterns have been shown to influence stress levels among nurses (Yinghao et al. 2023; Werke and Weret 2023).

Occupational stress has been found to be negatively correlated with both psychological resilience and overall quality of life (Diannita et al. 2024; Yan et al. 2022). In healthcare settings, the most commonly reported outcomes of occupational stress include burnout, turnover intention, reduced job satisfaction, anxiety and depressive symptoms (Okuhara et al. 2021). Research also indicates that elevated levels of occupational stress are associated with diminished psychological well‐being among nurses (Kurt et al. 2024; Shih et al. 2023). Beyond its impact on mental health, occupational stress adversely affects nurses' physical well‐being, disrupts familial and interpersonal relationships and undermines the quality of nursing care, thereby posing a threat to patient safety (Almino et al. 2024; Dartey et al. 2023; Mao et al. 2023; Zabin et al. 2023; Zhang et al. 2023). In addition to individual consequences, occupational stress impairs organizational adaptation functioning by hindering nurses' adaptation to the work environment (Baek and Han 2024), reducing job engagement (Yinghao et al. 2023) and increasing presenteeism (H. Jiang et al. 2021). As widely documented in the literature, occupational stress serves as a major determinant of turnover intention across various occupational groups (Freitas et al. 2023; N. Jiang et al. 2023; Lee et al. 2024; Lee and Kim 2020; Maharani and Tamara 2024; Piotrowski et al. 2022; Yang et al. 2021). Specifically, within the nursing profession, occupational stress is consistently identified as a significant predictor of turnover intention (Cetin Aydın et al. 2021; Bingöl et al. 2025; Piotrowski et al. 2022; Lee and Kim 2020; Saifan et al. 2024; Yang et al. 2021; Zhou et al. 2022). Despite the growing body of research on occupational stress and turnover, the potential effect of career decision regret on occupational stress remains underexplored. Furthermore, studies directly examining the relationship between career decision regret and turnover intention are limited, particularly in the nursing profession. To date, no empirical studies have identified the mediating role of occupational stress in the relationship between career decision regret and turnover intention. Addressing this gap, the present study investigates whether occupational stress mediates the relationship between career decision regret and turnover intention among practising nurses. The hypotheses are as follows:

*Occupational stress significantly predicts turnover intention among nurses*.

*Career decision regret significantly predicts turnover intention among nurses*.

*Occupational stress mediates the relationship between career decision regret and turnover intention among nurses*.

*Career decision regret mediates the relationship between occupational stress and turnover intention among nurses*.

**FIGURE 1:**
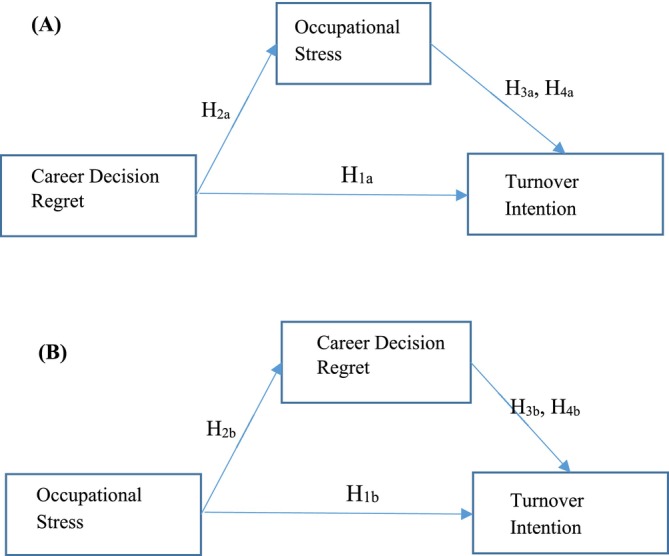
(A) Model 1: Mediation effect of occupational stress for the relationship between career decision regret and turnover intention. (B) Model 2: Mediation effect of career decision regret for the relationship between occupational stress and turnover intention.

The primary objectives of this study are threefold: (a) to examine the relationships among perceived career decision regret, occupational stress and turnover intention among nurses, (b) to investigate the mediating role of occupational stress in the relationship between career decision regret and turnover intention (Figure 1A) and (c) to explore the mediating role of career decision regret in the relationship between occupational stress and turnover intention within the context of hospital‐based nursing practice (Figure 1B). By identifying the key factors contributing to nurses' turnover intention, the findings of this study aim to provide valuable insights for hospital administrators and policymakers, supporting the development of strategies to enhance nurse retention.

## 2 Methods

### 2.1 Design

This study employed a descriptive cross‐sectional design.

### 2.2 Setting and Participants

The study was conducted with nurses working in hospitals across Türkiye, using an online data collection method. Inclusion criteria required participants to (a) be currently employed as a nurse in a hospital in Türkiye and (b) provide informed consent to participate in the study. Nurses who were in training or completing internships during the data collection period were excluded.

According to the 2023 Statistical Yearbook published by the Ministry of Health, there were 248 287 actively employed nurses in Türkiye, which constituted the target population for the study. The minimum sample size was calculated using the known‐population sampling formula: *n* = *Nt* ^2^ *pq*/[*d* ^2^(*N* − 1) + *t* ^2^ *pq*]. This calculation assumed a 95% confidence interval, a 5% margin of error and an expected proportion (*p*) of 0.50, to ensure the most conservative estimate and thus the largest required sample size. The study aimed to exceed this minimum requirement to enhance statistical power. A total of 524 nurses completed the online questionnaire during the designated data collection period. All responses were screened for completeness and quality. Twelve responses were excluded due to poor response quality (e.g., identical responses across all items such as ‘11111’ or ‘22222’), resulting in a final sample size of 512 nurses. Participants were recruited through convenience sampling.

### 2.3 Instruments

Data were collected using four instruments: the Nurse Information Form, the Career Decision Regret Scale, the Nurse Turnover Intention Scale and the Nurses' Occupational Stressor Scale (NOSS).

The Nurse Information Form was developed by the researchers to collect data on participants' personal characteristics (e.g., age, gender, education level and marital status) and professional characteristics (e.g., total years of professional experience, tenure at the current institution, current role or title, duration of duty, unit of employment and day/night shift status).

The Nurse Turnover Intention Scale, originally developed by Yeun and Kim (2013), was adapted into Turkish by Zeyrek et al. (2023). It consists of 10 items rated on a 5‐point Likert scale ranging from 1 (*strongly disagree*) to 5 (*strongly agree*). The scale is a single‐factor scale with no reverse‐coded items. Total scores range from 10 to 50, with higher scores indicating greater turnover intention. The original Cronbach's alpha was reported as 0.83. In the present study, the internal consistency was high, with a Cronbach's alpha coefficient of 0.927.

The NOSS was originally developed by Chen et al. (2020) to measure occupational stressors in clinical nursing. It was adapted and validated for Turkish use by Sarıalioğlu et al. (2022). The scale includes 21 items scored on a 4‐point Likert scale ranging from 1 (*strongly disagree*) to 4 (*strongly agree*). Scores range from 21 to 84, with higher scores indicating greater perceived occupational stress. The Turkish adaptation retained the original single‐factor structure. The Cronbach's alpha coefficient for the Turkish version was 0.82. In the present study, it was 0.937.

The Career Decision Regret Scale, originally developed by Brehaut et al. (2003), was adapted into Turkish by Erdurcan and Kırdok (2017), including validation and reliability analysis. The original scale uses a 5‐point Likert‐type rating ranging from 1 (*strongly agree*) to 5 (*strongly disagree*), with Items 2–4 reverse‐coded. For scoring, 1 is subtracted from each item score, the adjusted values are summed and the total is multiplied by 5, achieving a composite score between 0 and 100. The Turkish version is a self‐report measure designed to assess levels of career decision regret. It consists of five items rated on a 5‐point Likert scale ranging from 0 (*strongly disagree*) to 4 (*strongly agree*). In this version, Items 1, 3 and 5 are reverse‐coded. Scores are calculated by first reversing the specified items, summing all five item scores and then multiplying the total by 5. Final scores range from 0 to 100, with higher scores indicating greater levels of career decision regret. Score interpretation is as follows: 0–24 = ‘no regret’, 25–49 = ‘slight regret’, 50–74 = ‘moderate regret’ and 75–100 = ‘strong regret’. In the present study, the mean item score was used, with possible values ranging from 0 to 20. The Cronbach's alpha coefficient was 0.91 in the Turkish adaptation and 0.907 in this study.

### 2.4 Data Collection

Data for this study were collected between 1 November 2023 and 20 February 2024, using a web‐based questionnaire. Participants were recruited using the snowball sampling method, targeting nurses employed in hospitals across various regions of Türkiye. To enhance participation, a reminder message was sent to nonrespondents 15 days after the initial distribution of the survey.

### 2.5 Common Method Bias (CMB)

Procedural controls were implemented to minimize CMB. Clear and concise instructions were provided in the survey design, and anonymity of responses was ensured. In addition, complex or ambiguous items were avoided. To encourage honest and accurate responses, participants were explicitly informed that ‘there is no correct answer and all responses will be kept anonymous’. Two statistical techniques were employed to assess the presence of CMB. First, Harman's single‐factor test indicated that a single factor accounted for 41.10% of the total variance—below the commonly accepted threshold of 50%—indicating that CMB is unlikely to pose a serious threat (Harman 1967, 1976; Podsakoff 2003; Fuller et al. 2016). Second, confirmatory factor analysis (CFA) of a single‐factor model demonstrated poor fit indices: *χ* ^2^/df = 3.946, IFI = 0.826, TLI (NNFI) = 0.814, CFI = 0.825, GFI = 0.702 and AGFI = 0.666 (Bayram 2016). These values fall below recommended thresholds, further suggesting that a single‐factor model does not adequately represent the data. Together, the results from Harman's single‐factor test and the CFA support the conclusion that CMB is not a serious concern in the present study (Podsakoff 2003; Fuller et al. 2016).

### 2.6 Data Analysis

Statistical analyses were conducted using IBM SPSS Statistics Version 27 and AMOS Version 24 (IBM Corp., Armonk, NY, USA). To assess the normality of continuous variables, skewness and kurtosis coefficients were examined. All values were within the acceptable range of −1.5 to +1.5, indicating normal distribution. Cronbach's alpha coefficients were calculated to assess the internal consistency and reliability of each scale. Descriptive statistics were reported as frequencies and percentages for categorical variables and as means and standard deviations for continuous variables. For comparisons between two groups on continuous variables, independent samples *t* tests were used. One‐way analysis of variance (ANOVA) was performed for comparisons involving more than two groups. Following significant ANOVA results, the Scheffé post hoc test was applied to identify specific group differences. Pearson's correlation coefficient was used to examine relationships between continuous variables. Structural equation modelling (SEM) was performed to test the hypothesized relationships among variables. Two models were tested and presented in Figure 1. Model 1 examines the mediating role of occupational stress in the relationship between career decision regret and turnover intention, while Model 2 explores the mediating role of career decision regret in the relationship between occupational stress and turnover intention. Both models were analysed using the bootstrap method with 5000 samples and a 95% confidence interval. Statistical significance was determined at a two‐tailed *p* value of < 0.05.

### 2.7 Ethical Considerations

Ethical approval for this study was obtained from the Istanbul University Social Sciences and Humanities Research Ethics Committee (application date: 26 October 2023, number: 2215104). The study was conducted in accordance with the ethical principles outlined in the Declaration of Helsinki by the World Medical Association. To ensure anonymity, the questionnaire did not include any identifying information such as participants' names or contact details. Informed consent was obtained from all participants.

## 3 Results

### 3.1 Descriptive Characteristics of Nurses

A total of 512 nurses participated in the study, with a mean age of 34.79 ± 8.32 years. The majority were female (*n* = 425; 83%). Most participants were married (71%) and held a bachelor's degree or higher (75%). The average professional experience was 12.98 ± 8.09 years. Regarding work schedules, 40% worked exclusively day shifts. Participants were primarily employed in ward (37%) and operating room (33%) settings, with 58% working in the public sector. Detailed demographic and professional characteristics are presented in Table 1.

**TABLE 1:** Descriptive characteristics.

Variables	Statistics
Number of participants, *n* (%)	512 (100)
Age (years), mean ± SD	34.79 ± 8.32
Gender, *n* (%)	
Female	425 (83)
Male	87 (17)
Marital status, *n* (%)	
Married	364 (71.1)
Single	148 (28.9)
Educational status, *n* (%)	
High school	41 (8)
Associates	88 (17.2)
Bachelor's	307 (60)
Postgraduate	76 (14.8)
Unit, *n* (%)	
Ward	187 (36.5)
Operating room	167 (32.6)
Intensive care	93 (18.2)
Other	65 (12.7)
Years of professional experience (years), mean ± SD	12.98 ± 8.09
Years of experience in the unit (years), mean ± SD	10.34 ± 5.93
Work schedule, *n* (%)	
Day	207 (40.4)
Night	162 (31.6)
Shifts	143 (27.9)
Institution, *n* (%)	
Public	217 (42.4)
Private	295 (57.6)

Abbreviation: SD, standard deviation.

### 3.2 Scores on the Nurse Turnover Intention, Occupational Stressor and Career Decision Regret Scales

The mean score for nurse turnover intention was 34.56 ± 9.34, while the mean scores for career decision regret and occupational stress were 11.28 ± 5.34 and 58.94 ± 12.75, respectively. Significant positive correlations were found between turnover intention and both occupational stress (*r* = 0.811, *p* < 0.001) and career decision regret (*r* = 0.643, *p* < 0.001), indicating that higher levels of occupational stress and career decision regret are associated with increased turnover intention (Table 2). ANOVA revealed a significant difference in career decision regret based on education level (*F* = 3.395, *p* = 0.018), with nurses holding only a high school diploma reporting higher regret compared to those with higher educational qualifications. In addition, nurses working in public hospitals reported significantly higher levels of occupational stress (*t* = 4.404, *p* < 0.001), career decision regret (*t* = 2.956, *p* = 0.003) and turnover intention (*t* = 5.394, *p* < 0.001) than those working in private hospitals (Table 3).

**TABLE 2:** Scale scores and the level of relationships.

No.	Variables	Mean ± SD	Range	1	2
1	Occupational stress	58.94 ± 12.75	28–79	N/A	
2	Career decision regret	11.28 ± 5.34	0–20	0.458*	
3	Turnover intention	34.56 ± 9.34	13–47	0.811*	0.643*

*Note:* Pearson correlation test.

Abbreviations: N/A, not available; SD, standard deviation.

* *p* < 0.05.

**TABLE 3:** Occupational stress, career decision regret and turnover intention of nurses according to their demographic characteristics.

Variables	Category	*n*	Hemşire mesleki stresi	Mesleki karar pişmanlığı	İşten ayrılma niyeti
			Mean ± SD	Mean ± SD	Mean ± SD
Gender	Female	425	59.02 ± 12.78	11.39 ± 5.25	34.69 ± 9.16
	Male	87	58.56 ± 12.66	10.71 ± 5.78	33.90 ± 10.21
	*t*/*p* value		*0.305/0.760*	*1.079/0.281*	*0.673/0.502*
Marital status	Married	364	59.25 ± 12.84	11.23 ± 5.37	34.51 ± 9.37
	Single	148	58.18 ± 12.53	11.39 ± 5.30	34.68 ± 9.30
	*t*/*p* value		*0.861/0.390*	*0.296/0.767*	*0.184/0.854*
Educational status	High school	41	59.27 ± 12.75	13.73 ± 4.08	36.27 ± 8.16
	Associate	88	60.30 ± 12.17	11.44 ± 5.70	35.27 ± 9.06
	Bachelor's	307	59.25 ± 12.82	10.94 ± 5.27	34.46 ± 9.34
	Postgraduate	76	55.96 ± 12.90	11.12 ± 5.56	33.18 ± 10.19
	*F*/*p* value		*1.794/0.147*	*3.395/0.018* *	*1.190/0.313*
	Difference^a^			*1 > 3*	
Unit	Ward	187	59.01 ± 12.14	11.26 ± 5.35	34.79 ± 9.48
	Operating room	167	59.74 ± 12.50	11.37 ± 5.38	34.62 ± 9.27
	Intensive care	93	57.72 ± 13.60	11.08 ± 5.32	33.66 ± 9.26
	Other	65	58.45 ± 13.91	11.39 ± 5.37	35.02 ± 9.38
	*F*/*p* value		*0.537/0.657*	*0.069/0.977*	*0.381/0.767*
Total years of professional experience (years)	*r*/*p* value	512	*0.014/0.744*	*0.004/0.921*	*0.015/0.739*
Total years of experience in the unit (years)	*r*/*p* value	512	*0.010/0.814*	*−0.011/0.809*	*0.001/0.989*
Work schedule	Day	207	59.61 ± 12.78	11.21 ± 5.58	35.11 ± 8.76
	Night	162	57.58 ± 12.91	10.98 ± 5.44	33.32 ± 10.09
	Shifts	143	59.52 ± 12.48	11.71 ± 4.87	35.17 ± 9.20
	*F*/*p* value		*1.359/0.258*	*0.752/0.472*	*2.105/0.123*
Institution	Public	217	61.72 ± 11.35	12.08 ± 5.13	36.99 ± 7.60
	Private	295	56.90 ± 13.34	10.68 ± 5.43	32.77 ± 10.08
	*t*/*p* value		*4.404/< 0.001* *	*2.956/0.003* *	*5.394/< 0.001* *

Abbreviations: *F*, one‐way ANOVA; *r*, Pearson correlation; SD, standard deviation; *t*, independent sample *t* test.

^a^ Scheffé post hoc test.

* *p* < 0.05.

### 3.3 Mediating Model Analysis Results

#### 3.3.1 Model 1

Measurement Model 1, which included the variables of career decision regret (5 items), occupational stress (21 items) and turnover intention (10 items), was tested. Given the data demonstrated multivariate normality (multivariate kurtosis = −1.947, c.r.: −0.421), the covariance matrix was analysed using the maximum likelihood estimation method (Gürbüz and Şahin 2018). The model showed an acceptable fit according to key fit indices, confirming that the measurement model adequately fit the data (*χ* ^2^[591, *N* = 512] = 755.260; *p* < 0.01; *χ* ^2^/SD = 1.278; RMSEA = 0.023; CFI = 0.984; GFI = 0.928).

**FIGURE 2:**
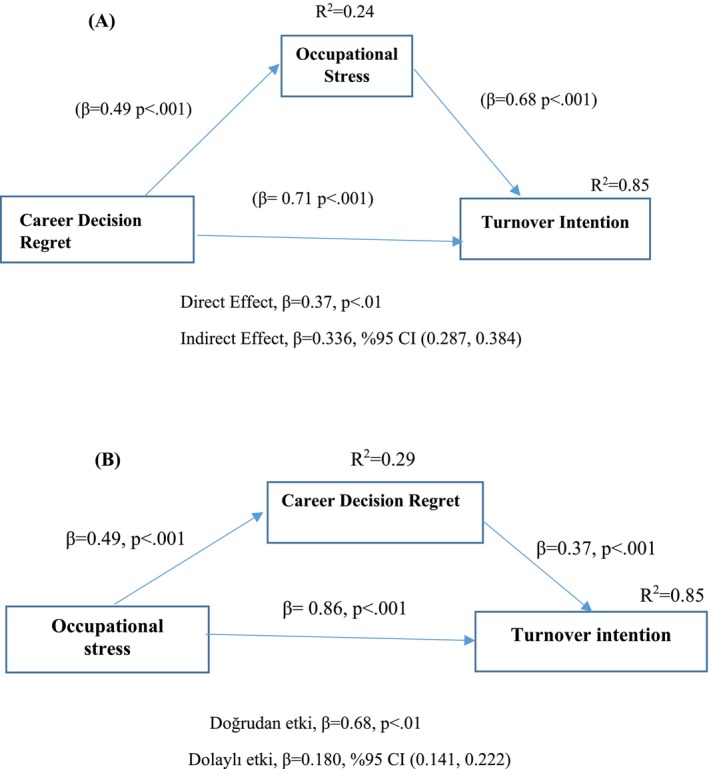
Results of structural equation model analysis. (A) Model 1: Mediation effect of occupational stress for the relationship between career decision regret and turnover intention. (B) Model 2: Mediation effect of career decision regret for the relationship between occupational stress and turnover intention. *Note:* Standardized beta coefficient values are reported. *R* ^2^ values indicate the explained variance. All models were controlled with demographics.

Following confirmation of the measurement model, the structural model with latent variables was tested to examine research hypotheses. Results are presented in Figure 2A. First, H1a (career decision regret → turnover intention) was supported, with career decision regret significantly predicting turnover intention (*β* = 0.71, *p* < 0.01). Next, H2a (career decision regret → occupational stress) was also supported, as career decision regret significantly predicted occupational stress (*β* = 0.54, *p* < 0.01). H3a (occupational stress → turnover intention) was tested within a mediation framework. Occupational stress significantly predicted turnover intention (*β* = 0.68, *p* < 0.01), confirming its mediating role. When occupational stress was included as a mediator, the direct path from career decision regret to turnover intention remained significant (*β* = 0.37, *p* < 0.01), indicating partial mediation. Together, career decision regret and occupational stress explained 85% of the variance in turnover intention (Table 4). The overall model fit remained excellent: *χ* ^2^(591, *N* = 512) = 755.260; *p* < 0.01; *χ* ^2^/SD = 1.278; RMSEA = 0.023; CFI = 0.984; GFI = 0.928; SRMR = 0.03. Bootstrap analysis with 5000 resamples confirmed the significance of the indirect effect of career decision regret on turnover intention, mediated by occupational stress (*β* = 0.336, 95% CI [0.287, 0.384]). These results support H4a, indicating that occupational stress mediates the relationship between career decision regret and turnover intention (Figure 2A).

**TABLE 4:** Results of the mediation model bootstrap analysis.

Latent variables	Model 1				Latent variables	Model 2			
	Outcome variables					Outcome variables			
	Occupational stress		Turnover intention			Occupational stress		Occupational stress	
	*β*	SE	*β*	SE		*β*	SH	*β*	SH
Career decision regret → turnover intention (c path)	—	—	0.71**	0.57	Occupational stress → turnover intention (c path)			0.86**	0.104
*R* ^2^	—	—	0.50		*R* ^2^	0.77			
Career decision regret → occupational stress) (a path)	0.49**	0.034	—	—	Occupational stress → career decision regret (a path)	0.49**	0.079	—	—
*R* ^2^	0.24		—	—	*R* ^2^	0.29		—	—
Career decision regret → turnover intention (c′ path)	—		0.37**	0.038	Occupational stress → turnover intention (c′ path)	—		0.68**	0.087
Occupational stress → turnover intention (b path)	—		0.68**	0.087	Career decision regret → turnover intention (b path)	—		0.37**	0.038
*R* ^2^	—		0.85		*R* ^2^	—		0.85	
Indirect effect (career decision regret → occupational stress → turnover intention)	—		0.33 (0.287, 0.384)		Indirect effect (occupational stress → career decision regret → turnover intention)	—	—	0.18 (0.141, 0.222)	

*Note:* The values in parentheses are the lower and upper confidence interval values. Bootstrap resampling = 5000.

Abbreviation: SE, standard error.

* *p* < 0.05.

** *p* < 0.001.

#### 3.3.2 Model 2

First, H1 (occupational stress → turnover intention) was tested. The results indicated that occupational stress significantly predicted turnover intention (*β* = 0.86, *p* < 0.01), supporting H1b. Next, H2b (occupational stress → career decision regret) was tested, and findings indicated that occupational stress significantly predicted career decision regret (*β* = 0.49, *p* < 0.01), supporting H2b.

Then, H3b (career decision regret → turnover intention) was examined by constructing a separate mediation model with career decision regret as the mediator. The analysis revealed that career decision regret significantly predicted turnover intention (*β* = 0.37, *p* < 0.01), supporting H3b. When occupational stress was included as an independent variable and career decision regret as a mediator, the direct path from occupational stress to turnover intention remained significant (*β* = 0.68, *p* < 0.01). Together, career decision regret and occupational stress explained 85% of the variance in turnover intention (Table 4). The path analysis showed a good model fit, with all fit indices exceeding commonly accepted thresholds: *χ* ^2^(591, *N* = 512) = 755.260, *p* < 0.01; *χ* ^2^/df = 1.278; RMSEA = 0.023; CFI = 0.984; GFI = 0.928; SRMR = 0.030. Bootstrap analysis with 5000 resampling iterations was used to evaluate the mediation effects, assessing significance through 95% confidence intervals. The indirect effect of occupational stress on turnover intention through career decision regret was significant (*β* = 0.336, 95% CI [0.287, 0.384]). These results indicate that career decision regret mediates the relationship between occupational stress and turnover intention, supporting H4b (Figure 2B).

## 4 Discussion

This study examined the relationships between nurses' personal and professional characteristics, career decision regret, occupational stress and turnover intention. A key finding is that nurses' turnover intention is positively correlated with both career decision regret and occupational stress. Furthermore, occupational stress emerged as a significant mediator in the relationship between career decision regret and turnover intention. In addition, career decision regret also served as a significant mediator between occupational stress and turnover intention.

The study further revealed that nurses working in public hospitals reported higher turnover intention compared to those in private hospitals. This finding is consistent with previous research noting similar trends (Polat et al. 2025). However, contrasting evidence exists, with some studies indicating that nurses in private hospitals exhibit greater turnover intention compared to those in the public sector (Freire and Azevedo 2023; Yeşilyurt et al. 2023; Yeşilyurt et al. 2021). Y. Kim and Kim (2024) suggest that the elevated turnover intention among nurses in public hospitals is largely attributed to challenging working conditions, such as inadequate nurse–patient ratios, excessive workloads and insufficient organizational support. On the other hand, nurses in private hospitals report lower turnover intention, which is considered to be associated with more favourable working conditions, including expedited recruitment processes and additional support services such as transportation, childcare and accommodation (Freire and Azevedo 2023; Yeşilyurt et al. 2021; Irviana and Asroriyah 2024; Hur and Abner 2024; Mihretie et al. 2022; Nigussie Bolado et al. 2023). A study in public hospitals across Türkiye identified significant nursing staff shortages, high workload ratios and an uneven workload distribution both between hospitals and within individual institutions (Özkan and Uydacı 2020). These factors contribute to a work environment that significantly elevates occupational stress among public sector nurses. In addition, research exploring nurses' perceptions of healthy work environments in public hospitals highlighted deficiencies in nurse workload management, career development opportunities, equitable wage policies and effective management and leadership (Yapıcı and Yürümezoğlu 2021). According to current legislation, private hospitals are required to maintain standardized patient‐to‐nurse ratios, except in specialized units such as intensive care. This discrepancy may contribute to the perception of workload among nurses in public hospitals. Supporting this, Yeşilyurt et al. (2021) found that performance‐based pay disparities and inconsistencies in additional compensation within public hospitals significantly influence nurses' decisions to leave their jobs. Notably, despite relatively better patient‐to‐nurse ratios in private hospitals, factors such as performance‐based pay disparities, job insecurity and low wages remain critical contributors to turnover intention among nurses in these settings (Yeşilyurt et al. 2021).

This study found that career decision regret significantly and positively predicts nurses' turnover intention. Career decision regret has been associated with career misfit, low professional and organizational commitment and decreased job satisfaction (Budjanovcanin et al. 2019; Doğanülkü and Şeker 2023; Akturan et al. 2025), all of which can indirectly increase the likelihood of turnover intention. However, some nursing studies with smaller sample sizes have reported a direct effect of career decision regret on turnover intention (Doğrusöz et al. 2022; Santra and Giri 2017). A broader review of the literature supports these findings. For instance, an international cohort study demonstrated a significant positive correlation between the intensity of care‐related regret among healthcare professionals and turnover intention, particularly when regret is accompanied by a high emotional burden (Cheval et al. 2019). Similarly, research conducted among cardiac physiologists in the United Kingdom revealed that career regret significantly predicts turnover intention (Budjanovcanin et al. 2019). In addition, studies examining the relationship between career‐related perceptions and turnover intention indicate that effective career fit reduces turnover intention (Sun et al. 2023). Given that career decision regret negatively impacts career fit (Doganülkü and Kirdök 2021), nurses experiencing regret may be less motivated to invest in their chosen profession, which could diminish their interest in new work‐related experiences and professional development opportunities. Career decision regret is recognized as a motivational emotion (Zeelenberg and Pieters 2007) that may compel nurses to consider career changes or leaving the profession altogether. The emotional weight of regret can lead nurses to psychologically distance themselves from their current roles as they seek alternative career paths, either within different healthcare settings or entirely different fields. Given the ongoing global nursing shortage, it is critical that healthcare managers and stakeholders proactively address career decision regret. Strategies such as improving working conditions, expanding career development opportunities and optimizing human resource practices may help reduce career decision regret and, consequently, lower turnover intention among nurses.

This study found that occupational stress positively affects nurses' turnover intention. Numerous studies across various fields have identified occupational stress as a significant predictor of turnover intention (Ekingen et al. 2023; Freitas et al. 2023; Liu et al. 2024). Although some research suggests that this relationship may not always be direct (Maharani and Tamara 2024), the present findings are consistent with a substantial body of literature emphasizing occupational stress as a critical factor influencing nurses' turnover intention (Cetin Aydın et al. 2021; Bingöl et al. 2025; Choi and Kim 2020; Piotrowski et al. 2022; Lee et al. 2024; Lee and Kim 2020; Saifan et al. 2024; Tariq et al. 2024; Yang et al. 2021; Zhou et al. 2022). A study conducted with primary healthcare professionals also identified occupational stress as a predictor of turnover intention (Ning et al. 2023). Similarly, a large‐scale prospective cohort study using actual turnover data found that high occupational stress levels significantly increased the likelihood of turnover (Kachi et al. 2020). Among the various sources of occupational stress, workload‐related stress, difficult interactions with patients' families and conflicts with managers or peers have been identified as major contributors to turnover intention among hospital nurses (Lee and Kim 2020). Occupational stress is also associated with reduced organizational commitment (Baek and Han 2024), which in turn raises turnover intention. Therefore, while reducing turnover intention remains a key goal, addressing the root causes of occupational stress is equally crucial.

This study also revealed that occupational stress mediates the relationship between career decision regret and turnover intention (Model 1). Although only a few studies have directly examined the association between career decision regret and turnover intention (Doğrusöz et al. 2022), the present findings provide novel insights into the mediating role of occupational stress. Career decision regret is often associated with poor career choices that do not align with an individual's interests, values or long‐term goals. This misalignment can lead to persistent dissatisfaction and elevated stress levels. Furthermore, missed career advancement opportunities can undermine self‐confidence, further increasing stress. For nurses, mistakes or errors at work—particularly those with serious consequences—can trigger intense feelings of guilt and regret, which may prompt ongoing self‐reflection and elevated stress. In addition, situations in which nurses perceive their actions as conflicting with personal values or face ethical dilemmas can generate considerable regret and stress. While career decision regret directly impacts turnover intention, it also exerts an indirect effect through occupational stress. Accordingly, career decision regret acts as a mediator in the relationship between occupational stress and turnover intention (Model 2). Occupational stress significantly influences both career decision regret and turnover intention (Park et al. 2023; Emiral et al. 2024; Akturan et al. 2025; Saifan et al. 2024; Bingöl et al. 2025). The findings of this study support the reciprocal relationship between occupational stress and career decision regret. Interestingly, the impact of career decision regret on turnover intention mediated by occupational stress is stronger than the effect of occupational stress on turnover intention mediated by career decision regret. This suggests that for nurses experiencing career decision regret, workplace stressors may amplify their regret, exacerbating turnover intentions. Therefore, career decision regret, when compounded by occupational stress, may lead nurses to convert their turnover intentions into actual turnover behaviour. Given the inherently stressful nature of nursing, targeted interventions such as vocational counselling, professional supervision and offering nurses greater autonomy in selecting their work units may effectively reduce career decision regret. Implementing these strategies could contribute to reducing turnover intentions and improving nurse retention.

### 4.1 Implications for Practice

Providing comprehensive career guidance and counselling, particularly before students begin their professional education, can significantly enhance alignment between individual interests and the demands of the nursing profession. Encouraging students to select careers that match their personal strengths and preferences may reduce the risk of future career decision regret. During nursing education, increasing students' awareness of the various nursing specialties by offering detailed information and guidance can empower them to make informed decisions about their specialization, better aligning their career paths with their goals.

Healthcare institutions can benefit from identifying nurses at risk of turnover by assessing their perceptions related to career decision regret and career satisfaction. Understanding these factors enables targeted interventions that address the root causes of turnover intention. Future research combining qualitative and quantitative methods would be valuable in revealing additional factors influencing nurses' turnover intentions. Integrating cognitive and behavioural strategies within the work environment can provide significant support to nurses. Stress management programmes such as counselling, peer support groups and mentoring can provide vital networks to help nurses cope with occupational stress and career‐related regrets. Encouraging nurses to proactively recognize and manage triggers of career decision regret through access to career counselling services may also reduce turnover intention by supporting necessary career adjustments.

Managers and policymakers hold a critical role in addressing professional dissatisfaction and promoting long‐term nurse retention. Sustaining professional engagement can be achieved through strategic interventions such as facilitating task transitions, providing certified training aligned with individual career interests, implementing formal recognition systems and creating supportive work environments that encourage retention. Ensuring fair allocation of resources—including equitable compensation, regulated working hours, optimal nurse‐to‐patient ratios, sufficient rest periods and flexible scheduling—can reduce perceptions of inequity that contribute to career decision regret and turnover intention.

Future studies should explore the underlying causes of career decision regret among nurses and investigate behavioural patterns that develop during and after the emergence of regret. In addition, the model proposed in this study could be applied across diverse nursing populations or extended by integrating other relevant variables.

### 4.2 Study Limitations

This study has several limitations that should be considered when interpreting the findings. First, the sample was limited to nurses employed in Türkiye, which may restrict the generalizability of the findings to broader or more diverse populations. Future studies should include larger, more heterogeneous samples across different countries and healthcare systems to enhance external validity. Second, the cross‐sectional design inherently limits the ability to establish causal relationships among career decision regret, occupational stress and turnover intention. Since data were collected at a single time point, only associations can be identified, not causal effects. The temporal sequence and potential confounding variables cannot be fully managed within this design. Therefore, the findings serve primarily to generate hypotheses rather than confirm causation. Future research should prioritize longitudinal or experimental designs to enable causal inferences and improve the reliability and generalizability of the findings. In addition, incorporating qualitative follow‐up studies could provide a deeper contextual understanding of these relationships. Third, the study relied on self‐reported questionnaires, which may introduce response bias and limit the objectivity of the findings. Self‐reports are susceptible to systematic biases such as social desirability, consistency bias, mood effects and perceptual distortions, potentially affecting all measured variables similarly. Fourth, the use of convenience and snowball sampling methods may limit the representativeness of the sample. Certain subgroups—such as healthcare professionals in rural areas or nurses working in diverse institutional contexts—may be underrepresented. Consequently, the findings may disproportionately reflect the experiences and perspectives of nurses employed in public hospitals or urban settings, limiting the broader applicability and completeness of the conclusions. Therefore, the impact of the sampling methods on participant distribution should be carefully considered, and these limitations must be acknowledged when interpreting the study findings. Finally, reliance on self‐reported measures introduces the potential for CMB, which poses an additional limitation. This bias arises when shared systematic error variance inflates, deflates or distorts the observed relationships between variables. To enhance accuracy and robustness in future research, it is recommended to incorporate additional data collection methods that complement self‐report instruments.

### 4.3 Recommendations for Further Research

To deepen the understanding of the development and consequences of career decision regret among hospital nurses, future studies should adopt comprehensive longitudinal research designs. The findings of the present study can serve as a foundation for developing targeted intervention strategies aimed at reducing nurses' turnover intention. Future research should also consider incorporating a broader range of variables—such as economic conditions, working hours, workload, psychological resilience and perceptions of organizational justice—to more comprehensively explore the multidimensional causes of occupational stress and career decision regret. In addition, replicating this study across diverse cultural contexts may provide valuable insights into the potential moderating role of cultural norms in the relationships among career decision regret, occupational stress and turnover intention.

## 5 Conclusion

This study examined the relationships among perceived career decision regret, occupational stress and turnover intention among nurses. The findings indicate that career decision regret significantly predicts nurses' turnover intention both directly and indirectly, with occupational stress serving as a partial mediator in this relationship. However, occupational stress was also found to significantly predict turnover intention both directly and indirectly through the mediating effect of increased career decision regret. In addition, the study revealed that nurses, particularly those employed in public hospitals, experience higher levels of occupational stress, career decision regret and turnover intention.

## Author Contributions

**Şehrinaz Polat:** conceptualization, data curation, funding acquisition, investigation, methodology, project administration, resources, software, supervision, validation, visualization, writing – original draft, writing – review and editing. **Aslı Yeşil:** conceptualization, data curation, funding acquisition, investigation, resources, software, supervision, validation, visualization, writing – original draft, writing – review and editing.

## Ethics Statement

This study was conducted in accordance with the Declaration of Helsinki, and all procedures involving human participants were approved by the Istanbul University Social Sciences and Humanities Research Ethics Committee (date: 26 October 2023, number: 2215104). Permission was obtained from the authors who adapted the scales used into Turkish. The options ‘I agree to participate in the study’ and ‘I do not agree to participate in the study’ were added to the voluntary consent form on the first page of the online survey form. Consent was obtained from participants who agreed to participate in the study. Only those nurses who checked the ‘I agree to participate in the study’ option were allowed to proceed to the other pages of the questionnaire form.

## Conflicts of Interest

The authors declare no conflicts of interest.

## Data Availability

The data that support the findings of this study are available from the corresponding author upon reasonable request.
